# Utilization of Marine Waste to Obtain β-Chitin Nanofibers and Films from Giant Humboldt Squid *Dosidicus gigas*

**DOI:** 10.3390/md19040184

**Published:** 2021-03-26

**Authors:** Gustavo Cabrera-Barjas, Cristian González, Aleksandra Nesic, Kelly P. Marrugo, Oscar Gómez, Cédric Delattre, Oscar Valdes, Heng Yin, Gaston Bravo, Juan Cea

**Affiliations:** 1 Unidad de Desarrollo Tecnológico, Parque Industrial Coronel, Universidad de Concepción, Concepción 3349001, Chile; g.bravo@udt.cl (G.B.); j.cea@udt.cl (J.C.); 2 Facultad de Ingeniería, Universidad del Bío-Bío, Concepción 4051381, Chile; crlgonza@alumnos.ubiobio.cl; 3 Department of Chemical Dynamics and Permanent Education, Vinca Institute of Nuclear Sciences—National Institute of the Republic of Serbia, University of Belgrade, Mike Petrovica-Alasa 12-14, 11000 Belgrade, Serbia; 4 Departamento de Físico-Química, Facultad de Ciencias Químicas, Universidad de Concepción, Edmundo Larenas 129, Casilla 160-C, Concepción 4070371, Chile; kmarrugo@udec.cl; 5 Carbon and Catalysis Laboratory (CarboCat), Department of Chemical Engineering, University of Concepción, Concepción 4030000, Chile; ogomez@udec.cl; 6 Clermont Auvergne INP, Université Clermont Auvergne, CNRS, Institut Pascal, F-63000 Clermont-Ferrand, France; cedric.delattre@uca.fr; 7 Institute Universitaire de France (IUF), 1 rue Descartes, 75005 Paris, France; 8 Centro de Investigación de Estudios Avanzados del Maule (CIEAM), Vicerrectoría de Investigación y Postgrado, Universidad Católica del Maule, Talca 3460000, Chile; ovaldes@ucm.cl; 9 Dalian Engineering Research Center for Carbohydrate Agricultural Preparations, Liaoning Provincial Key Laboratory of Carbohydrates, Dalian Institute of Chemical Physics, Chinese Academy of Sciences, Dalian 116023, China; yinheng@dicp.ac.cn

**Keywords:** Humboldt giant squid, β-chitin, nanofibers, characterization, films

## Abstract

β-chitin was isolated from marine waste, giant Humboldt squid *Dosidicus gigas*, and further converted to nanofibers by use of a collider machine under acidic conditions (pH 3). The FTIR, TGA, and NMR analysis confirmed the efficient extraction of β-chitin. The SEM, TEM, and XRD characterization results verified that β-chitin crystalline structure were maintained after mechanical treatment. The mean particle size of β-chitin nanofibers was in the range between 10 and 15 nm, according to the TEM analysis. In addition, the β-chitin nanofibers were converted into films by the simple solvent-casting and drying process at 60 °C. The obtained films had high lightness, which was evidenced by the CIELAB color test. Moreover, the films showed the medium swelling degree (250–290%) in aqueous solutions of different pH and good mechanical resistance in the range between 4 and 17 MPa, depending on film thickness. The results obtained in this work show that marine waste can be efficiently converted to biomaterial by use of mild extractive conditions and simple mechanical treatment, offering great potential for the future development of sustainable multifunctional materials for various industrial applications such as food packaging, agriculture, and/or wound dressing.

## 1. Introduction

In Chile, near 46,000 ton/month of Humboldt squids (*Dosidicus gigas* Wild) are caught for human consumption. According to the annual fishing statistics reports from 2019, the Chilean fishing quota of the giant squid of Humboldt is 200,000. During its industrial processing, the squid pen is generated as a by-product, which is currently used as a fish flour extender, which presents low value-added processing. Hence, there is an urgent need for the development of new strategies to convert marine waste into value-added materials from an economical point of view. One of the approaches is the extraction of components from waste and by-products of the fishery industry and their conversion to value-added materials [1,2,3]. Particular attention is given to squid pens because they are a valuable source of β-chitin and protein.

Generally, chitin or poly (β-(1→4)-*N*-acetyl-d-glucosamine) is a natural polysaccharide derived from large numbers of living organisms [4,5,6]. It is the second most abundant natural polymer, next to cellulose. In the native state, chitin occurs as ordered crystalline microfibrils, which are structural components in the exoskeleton of arthropods or fungal cell walls. Depending on its source, chitin exists as two crystalline allomorphs, namely in *α*- and *β*- form. The *α*-chitin isomorph is by far the most abundant because it can be found in the exoskeletons of crustaceans and cell walls of fungi and yeasts. On the other hand, *β*-chitin is rare in nature and is found in association with proteins in squid pens and the tubes of some worms [7,8]. The *α*-chitin was mainly studied due to vast abundance; however, this allomorph is highly crystalline and insoluble in aqueous or common organic solvents, limiting its application. In addition, when it comes to the extraction process of α-chitin from crustaceans, the general procedure requires a first demineralization process using a dilute mineral acid for several hours to separate calcium carbonate. Then, a deproteinization under harsh conditions (high temperature and longtime of treatment) is required to extract proteins. The amount of extracted chitin at these conditions is approximately 20%. On the other hand, the extraction of β-chitin from squid pens does not require harsh demineralization conditions since squid pens contain only up to 5% of minerals. The yield of β-chitin extracted from squid pen is usually around 50% [9]. It is known that β-chitin is less crystalline allomorph and more soluble in organic solvents, making this type of chitin more reactive. Moreover, due to parallel chain arrangements, the β-chitin-based material possesses higher mechanical stability than α-chitin based material [10]. Hence, squid pen presents a beneficial source of chitin from the environmental and economical point of views, where the extraction process can be performed at mild conditions, with reduced chemical and energy consumption, and higher reactivity of obtained biopolymer, in comparison to the α-chitin obtained from crustaceans.

The present work aims to obtain and characterize β-chitin microfibers (MF) and nanofibers (NF) from squid pen Dosidicus gigas. Up to date, there are several studies in the literature for the extraction of β-chitin from different species of squid pens [11,12,13]. However, the conversion of β-chitin microfibrils into β-chitin nanofibrils and their detailed characterization is a new topic, and only a few papers in the literature can be found. Suenaga et al. studied the Star Burst system (wet pulverization technique under high pressure) to obtain β-chitin nanofibers in distilled water and under acidic conditions [7,14]. Ifuku et al. also used the Star Burst system to obtain chitin nanofibers [15]. On the other hand, numerous methods were used to obtain α-chitin nanofibers such as ultrasonication [16], grinding [17], or dynamic high-pressure homogenization under acidic conditions [18]. This work will present a new and simple method to process β-chitin nanofibers at a bench-scale of low energy and acid consumption. Namely, β-chitin microfibers were dispersed in an acidic aqueous solution (pH 3) and converted into nanofibers by pass-through a collision machine. SEM, TEM, and XRD characterized the obtained nanofibers. It is important to underline that obtained β-chitin nanofibers can be directly shaped into transparent films by a simple drying method in the oven. Hence, chitin nanofibers-based films were also characterized along with the raw material and nanofibers by different physicochemical and microscopic techniques. These chitin nanofibers can find applications in food, cosmetics, biomaterials, agriculture, electronics, adhesive, and biomaterials areas [19,20,21,22,23,24]. 

## 2. Results and Discussion

### 2.1. Squid Pen and β-Chitin Isolated Composition

It is well known that the chemical composition of squid pens depends on species; therefore, ash content can vary from 0.02–2.4%, protein content from 43–76%, and chitin content from 25–50% [19]. The chemical composition of squid pen and isolated *β*-chitin is presented in Table 1. It has been proven that squid *D. gigas* pen is an excellent source of chitin (30.2% by dry weight). Similar chitin content of squid pens was obtained in the literature for various species: *Todarodes pacifica* (26%) [19], *Illex argentinus* (31%) [9], *Loligo vulgaris* (31%) [25], and *Sepioteuthis lessoniana* (40%) [26].

The average ash, lipid, and protein contents of the squid pen *D. gigas* were 1.8%, 0.40%, and 67.5%, respectively. The ash content of squid pen *D. gigas* is similar to the one for squid pen *Loligo* sp. (1.9%) [13] and *Loligo vulgaris* (1.7%) [27]. The squid pen *D. gigas* has been shown to be a good source of protein (68%), and this is also observed for the squid pen *Loligo Chenisis* (57%) [12], *Illex argentinus* (64%) [11], and *Todarodes pacifica* (75%) [19].

The extraction of β-chitin from squid pen was performed by demineralization in HCl (1 M) and deproteinization in NaOH (1 M). The yield of extracted chitin was 39.4%. Other authors reported similar yields for the extraction of *β*-chitin from squid pens (35–42%) [12,13]. It can be noticed that inorganic compounds are significantly removed (<1%) during extraction of β-chitin, which is evidenced by reduced ash content. Regarding the DA of obtained β-chitin, it was higher than reported from Loligo Chenisis squid pen (80.3%) [12], but lower than reported for *Loligo vulgaris* (100%) [27].

Molecular weight determination of chitin samples is quite complex because it requires sophisticated analytical capabilities. Hence, simple indirect methods to compare the macromolecules’ molecular weight are used. Among these, viscosity methods are very cheap and useful. The reduced viscosity parameter is measured in a dilute solution of macromolecules and can provide information on its shape, flexibility, and (for no spherical particles) molar mass of macromolecules. It can be used as a comparison value because of its concentration-dependent nature. The obtained η_red_ value for the β-chitin sample was high (2648 mg/mL), suggesting that this macromolecule can have a high molecular weight, and the employed extraction method does not lead to an extensive chain degradation. This result agrees with previously reported values of reduced viscosity for other crustacean and squid pen β-chitin [28].

### 2.2. Physicochemical Characterization of β-Chitin

#### 2.2.1. ^13^C CP/MAS Solid-State NMR Analysis

**Figure 1:**
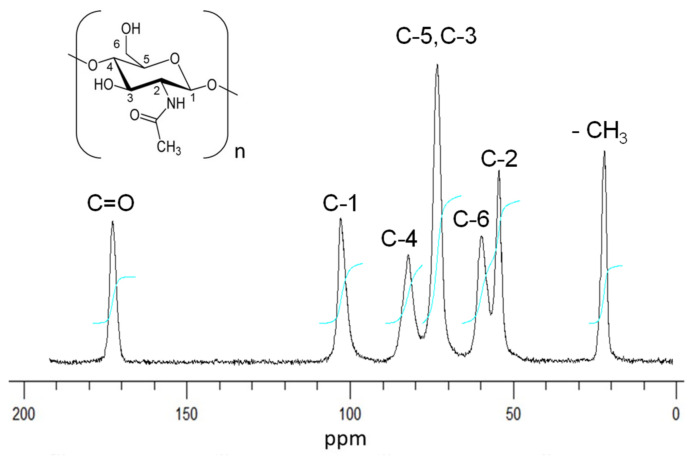
^13^C CP/MAS NMR spectra of *D. gigas* β-chitin.

The degree of deacetylation of isolated β-chitin was determined by ^13^C CP/MAS solid-state equipment and spectrum is presented in Figure 1. Seven signals were detected that were ascribed to the eight carbon atoms of the *N*-acethylglucosamine repetitive unit, which appear at the following chemical shifts: δ = 173.3 ppm (C=O), 104.5 ppm (C-1), 85.2 ppm (C-4), 75.7 ppm (C-3 and C-5), 59.9 ppm (C-6), 55.8 ppm (C-2), and 23.2 ppm (CH_3_). The C=O signal appears as a sharp and symmetric profile. The C-3 and C-5 signals merge into a single resonance centered at 75.6 ppm, which is a characteristic pattern for *β*-chitin. All of these signals are typical for *β*-chitin and confirm its conformational state. The relative intensities of the resonance of the ring carbon (IC1, IC2, IC3, IC4, IC5, IC6) and CH_3_ group (I_CH3_) were compared in order to calculate the degree of acetylation (DA) value. It was found that the DA of β-chitin isolated from *D. gigas* was 96.4%. Generally, DA of chitin obtained from different species of squid pen varies from 80 to 98%, depending on the different extraction parameters: presence or absence of demineralization process, time, and temperature of operated demineralization and deproteinization process as well as the concentration of solutions used for these processes [11,24,25,29,30]. The high content of DA obtained in this work indicates that mild extraction conditions for the extraction of chitin are used, thus preserving the native structure of β-chitin.

#### 2.2.2. FTIR Analysis

**Figure 2:**
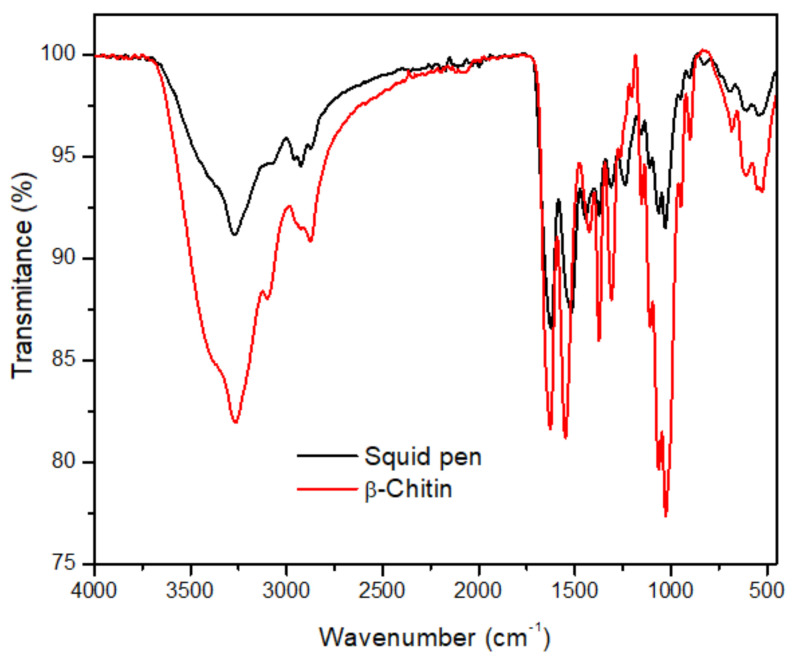
FTIR analysis of *D. gigas* squid pen and isolated β-chitin.

The FTIR spectra of squid pen and isolated β-chitin are presented in Figure 2. The main difference between spectra (e.g., the bands intensity, band shifting, and some signals overlapping) is due to the raw material protein contents, as previously discussed (see Section 2.1). Hence, the main focus will be given to the discussion of the β-chitin spectra. The β-chitin FTIR spectra patterns are similar to those reported in the literature [7], suggesting that good chitin quality had been obtained. An exhaustive examination of the spectrum revealed that the spectra had the characteristic bands of polysaccharides. Namely, the wide area in the region between 3600 and 3000 cm^−1^ was detected: bands at 3472, 3326, and 3101 cm^−1^, which correspond to the stretching vibration of –OH and –NH groups (*ν*OH, *ν*^as^ NH, and ν^s^ NH), respectively. The first band belongs to –OH groups involved in hydrogen bonds (O-6-H ···O=C and O-3-H·· ·O-5). The other two bands are due to C=O ·H-N intermolecular hydrogen bonding and H bonded –NH groups, respectively. The chitin characteristic bands related to –CH groups stretching vibration appears at 2962 cm^−1^ (ν^as^_CH3_), 2929 cm^−1^ (ν^s^_CH2_), and 2880 cm^−1^ (ν^as^_CH3_). Moreover, the spectra also feature characteristic bands at 1627, 1548, and 1313 cm^−1^ that are describing the vibrations of Amide I (primarily C=O stretch), Amide II (bending vibration of N-H), and Amide III (ν _C-N_ + δ _NH_) groups, respectively. The presence of one single peak in the region between 1600 and 1670 cm^−1^ confirms the presence of *β*-chitin. It is known that the carbonyl oxygen of the acetamide group formed intermolecular hydrogen bonding between the primary –OH and –NH_2_ groups, forming a two-dimensional hydrogen bonding network in a plane perpendicular to the pyranosyl plane. The spectra of β-chitin also revealed two additional bands of –CH group deformations around 1425 (δ _CH2_) and 1375 cm^−1^ (δ _CH_ + δ _C-CH3_), and a greater number of narrower bands in the region between 1200 and 1032 cm^−1^, related to C–O–C and C–O stretching vibrations. Another characteristic marker is the CH deformation of the β-glycosidic bond. This band appears in β-chitin at 895 cm^−1^ (γ _CH_, C1 axial of β-linkage). Therefore, FTIR analysis confirmed that the isolated biopolymer is *β*-chitin. This result is in agreement with the results obtained by ^13^C CP/MAS NMR analysis, where the crystalline structure of identified chitin was proven.

#### 2.2.3. Thermogravimetric Analysis

**Figure 3:**
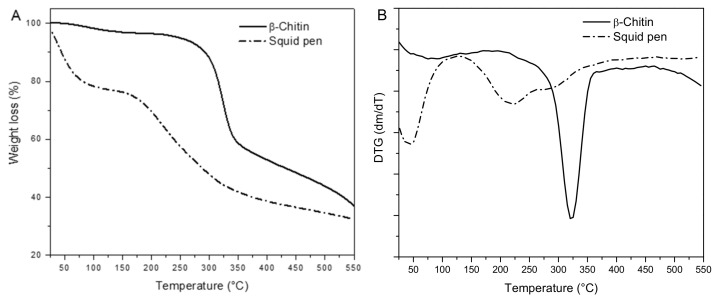
Thermogravimetric (TG) curves. (**A**) TG and (**B**) derivate thermogravimetric (DTG) of *D. gigas* squid pen and isolated β-chitin.

**Figure 4:**
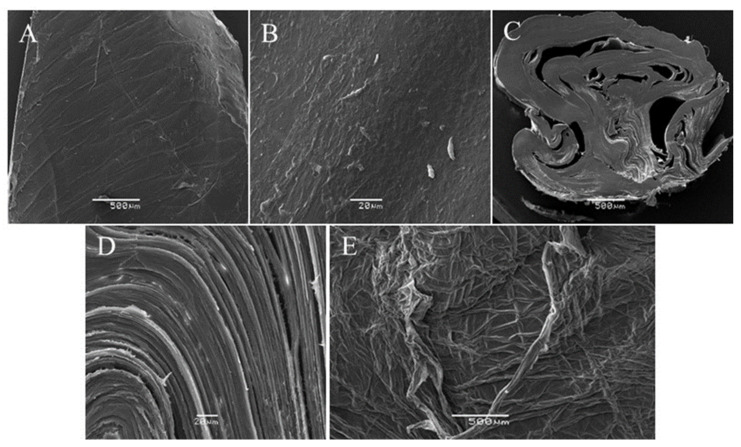
SEM micrograph of *Dodeciduos gigas* gladius blade region surface (**A**,**B**), cross-section view of gladius center (**C**,**D**), and β-chitin surface (**E**).

Thermograms of squid pen and isolated β-chitin are shown in Figure 3. The thermal analyses of squid pen and isolated β-chitin were undertaken in the interval from 20 to 550 °C. The thermogravimetric (TG) and its derivate (DTG) curves are presented in Figure 4. The squid pen’s overall decomposition process (66% weight loss) consists of three degradation steps, which are reflected as three peaks in the DTG curve. The first degradation stage (25–126 °C), with the maximum degradation at 46 °C is due to the water evaporation in the squid pen, and it accounted for a weight loss of 23%. In the range of 126–264 °C, the second weight loss can be attributed to the depolymerization and degradation of the squid pen proteins that wrap chitin fibers. This stage is the main difference between squid pen and isolated β-chitin thermograms. A third stage between 264 and 550 °C and maximum degradation at 294 °C is caused by the degradation, pyrolysis, vaporization, and elimination of the volatile products of remaining proteins and the chitin chain [31].

On the other hand, the thermogram corresponding to the decomposition of *β*-chitin showed two-stage degradation step mechanisms. In the first stage, 3.6% of weight loss occurred due to the water evaporation, followed by a second degradation step with the maximum decomposition temperature at 325 °C, which describes the polymer chain degradation. In this stage, the associated mass loss was 59.4%. This is a complex process where simultaneous monomer dehydration, chain scission, and thermal decomposition of glucosamine and N-acetylated units occurs [4]. Consequently, chain packing is loosened. Hence, a relatively small amount of heat is required for its degradation [32]. This result agrees with those found during thermal degradation of β-chitin isolated from *Loligo vulgaris gladii* [33]. Finally, the results are summarized in Table 2.

#### 2.2.4. SEM Analysis

The surface structure of squid pen and isolated β-chitin were studied, and their morphology is presented in Figure 4. The SEM micrographs showed that the squid gladius blade region had a rough and fibrous surface morphology (Figure 4A,B). The micrographs from the cross-section view of the same material (Figure 4C,D) showed that the squid gladius blade was composed of large microfiber (5–10 µm diameter) aggregates. Similar fibrous morphology was observed in the gladius from other squid species [33]. It is known that proteins and β-chitin chemically form squid gladius [34]. Moreover, the squid gladius’s hierarchical structure was reproduced at the nanoscale and it was found that β-chitin nano-crystallites were wrapped in a protein layer to form nanofibrils. These were the building blocks of 200 nm sized nanofibers. The nanofibers aggregated into 2 µm, 10 µm, 100 µm, and 500 µm thick fibers, respectively, which eventually formed the gladius.

In the case of β-chitin surface analysis (Figure 4E), a highly fibrous structure was observed. This could be associated with the preparation methodology, which allowed for the removal of the protein that surrounded the chitin fibers by a smooth alkaline treatment. Such morphology has been associated with the chain arrangement in β-chitin crystals that are packed in a parallel manner. In this polymorphic structure, the carbonyl oxygen of the –NHCOCH_3_ group is involved in intermolecular hydrogen bonding between the primary –OH and –NH groups, forming a two-dimensional hydrogen bond network in a plane perpendicular to the chitin pyranosyl plane [35].

### 2.3. Nanofibrillated β-Chitin Characterization

#### 2.3.1. Viscosity

**Figure 5:**
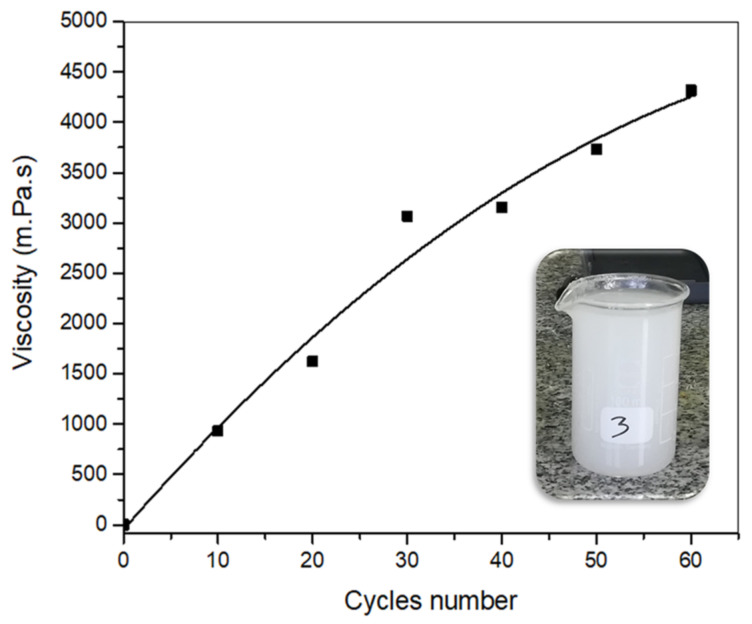
Viscosity of NFCh gels (pH 3) obtained after different cycle numbers (collision time) and picture of gel obtained after 60 cycles.

As a result of the fibrillation process, the macromolecular fiber entanglement is separated into individual fibers. Then, a conversion from a low-viscosity chitin suspension to a high-viscosity gel network formation occurs. Viscosimetry is a cheap and widespread technique used to measure the polysaccharides solution viscosity at an industrial scale. Hence, it can be used to follow the progress of chitin defibrillation in acidic aqueous media. Results from viscosity measurements of nanofibrillated chitin gels obtained after different cycle numbers (pass through collision machine) are presented in Figure 5. It can be observed that the 1 wt% chitin suspension viscosity increased along with the cycle number from 10 to 60. A white gel with good stability at low temperatures (4 °C) can be obtained this way. It is known that a high aspect ratio (length/diameter) or longer and thinner chitin nanofibers promotes the entanglement possibility within nanofibrils, improving the shear viscosity of the suspension.

#### 2.3.2. TEM Analysis

**Figure 6:**
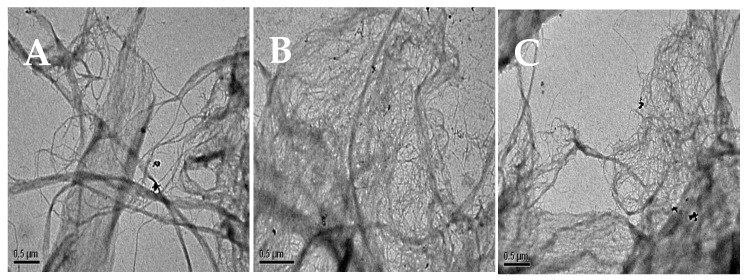
TEM micrograph of nanofibrillated β-chitin after different fibrillation cycles: (**A**) 20 cycles, (**B**) 40 cycles, and (**C**) 60 cycles.

TEM analysis was performed to study the effect of the fibrillation process on the β-chitin hierarchical structure. For this purpose, dilutions of the fibrillated gels obtained after different fibrillation cycles were studied. Results are presented in Figure 6. All samples were analyzed at a gel concentration of 0.01 wt% due to the high concentration of originally obtained nanofibers. It can be observed that after 20 cycles (collision time), there was still the presence of microfibrils in the suspension gel. The width (diameter) of β-chitin nanofiber was in the range of 100–250 nm and it did not disperse uniformly. Thicker fibers in the range of 600 nm could also be detected.

Conversely, from 40 cycles onward, the processing of nanofibers was improved, and the decrease in nanofiber diameter within an increase of cycles through collision equipment from 40 to 60 was noted. The obtained β-chitin nanofibers after 60 cycles had a mean diameter in the range of 10–15 nm. A similar nanofibril diameter (6.4–6.9 nm) was previously observed for β-chitin nanofiber prepared from *Loligo vulgaris* at different pH levels [36] and from *Todarodes pacificus* (5–10 nm) [5].

### 2.4. Nanofibrous β-Chitin Film Preparation and Characterization

**Figure 7:**
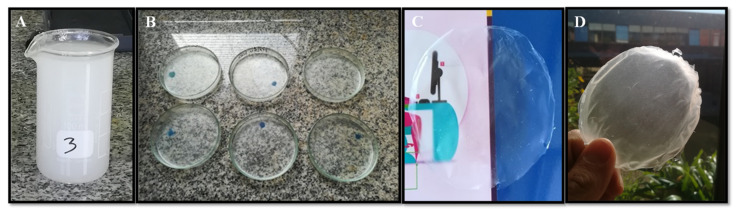
Camera photography of the process preparation of NF β-chitin films by the solvent casting method. Chitin nanofibrillated gel (1 wt %) (**A**), casting films with different amount of gels 1 wt% (15, 20, 30mL from left to right) (**B**), and resulting films with different thickness: 0.03 mm (**C**) and 0.05 mm (**D**).

One of the main drawbacks of chitin application is their very low solubility in common organic solvents and their solubility in complex solvent mixtures. It makes processing difficult to obtain novel materials with industrial applications such as films, sponges, nano/microparticles, fibers, aerogels, and composite materials. Recently, ionic liquids and other alkali-based solvents have been proposed to overcome this issue [37,38,39,40]. However, in all cases, there is still a need for solvent removal. Nanofibrillated chitin gels obtained in an acidic aqueous solution (pH 3) offer a unique possibility to process chitin-based materials in different forms and shapes for different applications. For instance, it can be used as reinforced material for biocomposite preparation [41] and it can be readily processed into films, hydrogels, or aerogels [8,16,42]. In the present work, the preparation and characterization of nanofibrillated chitin films are presented (Figure 7). This is only one example of their feasibility to be handled. This process takes advantage of chitin nanofibrils’ natural tendency to self-assemble in aqueous media to form *β*-chitin fibers [36].

#### 2.4.1. X-ray Diffraction (XRD) Analysis

**Figure 8:**
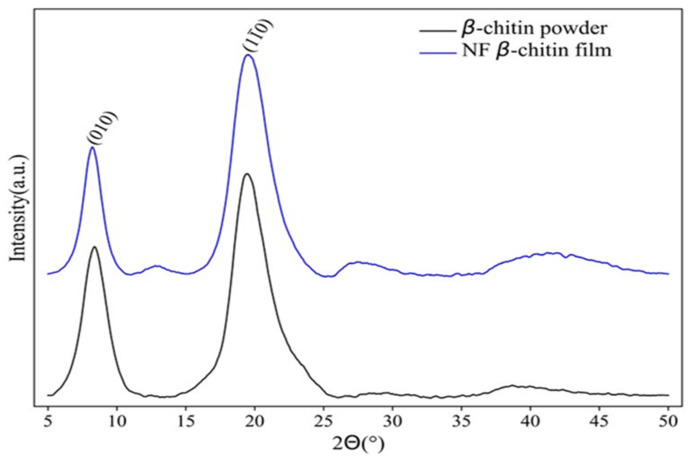
X-ray diffraction (XRD) diffractogram of β-chitin microfibril powder (black line) and film samples (blue line).

The crystalline structure of the β-chitin, nanofibrillated β-chitin film, and nanofibrillated β-chitin foam was analyzed by x-ray diffraction. The corresponding diffractograms are shown in Figure 8.

It is important to underline that β-chitin nanofibrils in films showed a similar pattern as β-chitin microfibril powder (see Table 3). This means that the β-chitin crystal structure is preserved after mechanical treatment. The diffractogram of β-chitin powder displays two broad peaks at 2θ = 8.22° (inter-sheet distance: 10.76 Å) and 2θ = 19.32° (4.59 Å), which corresponds to the crystal plane [010] and [1$\bar{1}$0] reflection values, respectively [7,32]. The crystallinity index of *β*-chitin microfibril powder was 69.7%, which indicates a high crystalline structure. This value was higher than that obtained for chitin from squid pen *L. vulgaris* (58%) [30], similar to the one found for squid pen of the same specie (67%) caught in the Northern Hemisphere [43], and slightly lower than for chitin obtained from squid pen *Illex argentinus* (75%) [11]. The crystallinity index of the β-chitin nanofibrils film is slightly lower than that for the β-chitin microfibril powder. Moreover, the peak of the [1$\bar{1}$0] plane of NF β-chitin shifted to a higher value, indicating a decrease in *d*-spacing (see Table 3). The *d* spacing values found for the β-chitin powder agreed with those previously reported (10.0 and 4.5) for this specie [43]. The D_ap_ values in the perpendicular direction of the [010] plane were 6.0, and 4.7 nm for the original squid pen β-chitin and the nanofibers, respectively. This is an indication that the crystallite size in the [010] plane region decreases during fibrillation. These values are higher than those found in similar products obtained from the squid pen of *T. pacificus* (4 and 3.5 nm) [44]. According to these authors, the specific surface areas highly increased during the fibrillation process, and disordered regions in the XRD analysis were explained by the contact of nanofibers with the water. This could explain the lower crystallinity and crystallite size reduction in the NF β-chitin samples. All of these results confirm the disintegration of β-chitin powder into nanofibrous β-chitin during the mechanical treatment.

#### 2.4.2. SEM Analysis of Films

**Figure 9:**
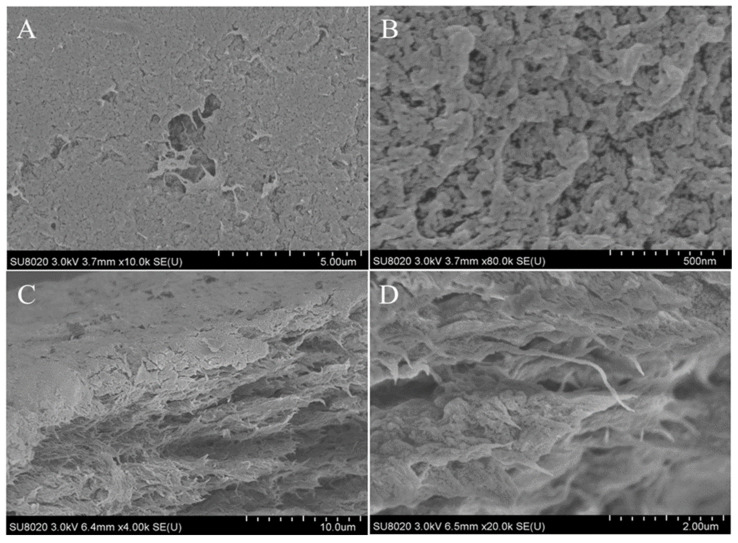
SEM micrographs of cryo-fractured NF β-chitin film’s surface (**A**,**B**) and the cross-section (**C**,**D**).

In order to study the morphology of the obtained chitin nanofiber films, SEM analysis was performed. The results are presented in Figure 9. The surface of the nanofibrillated chitin film had a non-continuous rough and fibrous morphology (Figure 9A). At higher magnification (Figure 9B), a fiber collapse shape with some porosities could be seen. The side view (cross-section) depicts the films’ inner fibrous appearance (Figure 9C,D) and shows a formed three-dimensional interconnected network under the surface. It is important to note that chitin films seem to be self-assembled in nanofiber sheets. These micrographs confirm the high defibrillation reached during chitin processing at the bench scale.

#### 2.4.3. Mechanical, Colorimetric, and Swelling Degree Properties

Mechanical properties of the β-chitin nanofiber films with different thicknesses were evaluated and are presented in Table 4. As the thickness of film increased from 0.02 to 0.05 mm, the tensile strength of the film increased significantly (from 3.9 to 17.4 MPa), whereas elongation at break decreased (from 3.5 to 1.7%). The Young’s modulus of β-chitin films also increased from 0.9 to 1.1 GPa. The mechanical parameters obtained in this work were in the same range as the data from the literature for other chitin films. Namely, Kaya et al. showed that E and TS values of α-chitin films extracted from *B. giganteus* cockroach dorsal pronotum (thickness 0.07 mm) were 492 MPa and 11.7 MPa, respectively, and the elongation at break was 3.3%. These parameters for chitin films extracted from cockroach wings (thickness 0.01 mm) were 476 MPa, 6.2 MPa, and 2.2%, respectively [45]. Other authors reported that nanofibrillated α-chitin films (thickness 0.06 mm) from crab showed E and TS values of 3.3 GPa and 32 MPa, respectively. However, they found a lower e value (1.3%) [46]. Furthermore, Ifuku et al. informed that NF α-chitin films (thickness 0.06 mm) had a Young’s modulus of 2.5 GPa and a TS of 44 MPa, respectively [47]. It is worth noting that those films had higher thicknesses than those from this work, affecting the final mechanical properties. Other authors have studied β-chitin nanofiber preparation from squid pen, and they found that the resulting film’s (thickness 0.045 mm) mechanical properties (E = 2.8 GPa, TS = 19 MPa) changed with the number of passes during preparation [48]. The β-loss in chitin nanofiber crystallinity accounted for the decrease in TS observed from 10 to 50 passes, while the E values remained almost constant. In these previous works, the fiber cross-sectional width was near half (3–9 nm) that obtained in the present work, and it is known that thinner and larger nanofibers show higher mechanical properties. In general, the mechanical properties of NF films are influenced by chitin chemical composition (e.g., Mw, CI, DA) and nanofiber structure (length/width ratio), which in turn depends on the biopolymer source along with the isolation procedure, and the NF preparation methods [43,49,50]. Other properties that could affect the nanofibrillated chitin film mechanical properties are the porosity, protein content, thickness, preparation method, and NF orientation [51,52,53]. Finally, these NF were prepared at a laboratory scale under controlled conditions. Considering this fact, the obtained results from this work at a bench-scale are auspicious if this cheaper process would be further scaled.

In order to check the colorimetric parameters of the obtained chitin nanofiber films and their changes within the different thickness of films, the CIELAB color system test was applied. According to the parameters presented in Table 4, the films had a high L* value, which means that they had high lightness. The negative a* value and positive b* value indicated that films were green-yellowish. ΔE* is a parameter that can show the difference in the color between the samples. In this paper, the color differences among chitin nanofiber films of different thicknesses were tested toward the blank white surface. As can be seen, the ΔE* value was not significantly different for two different chitin nanofiber films, which means that there was no significant difference in their color. Moreover, the values of ΔE* were above 3, indicating that color change is visually perceivable.

The swelling degree of β-chitin nanofiber films was measured at three different pH values (4, 6, and 8). As shown in Table 4, the swelling degree of the tested films slightly decreased when the pH increased from pH 4 to pH 6. However, these changes were not significant. In addition, there were no significant changes in the swelling degree value between films of different thicknesses. On the other hand, under alkaline conditions, all swelling degree of all tested films decreased in a range of 7–12%. This result is expected since it is known that the pK0 of chitosan is around 6–6.5. At pH 4, there are positively charged glucosamine units in chitin chains, and hydrogen bonding with the molecules of water occurs. However, due to the high DA of β-chitin, the low amount of free-glucosamine units in the backbone are not enough to provoke a significant change in the degree of swelling of those films. As the pH increases and switches to the alkaline environment, the amine groups from chitin become deprotonated, which leads to repulsion interaction between the chitin chain and water molecules and reduced the swelling degree.

Finally, the biodegradable chitin nanofibers obtained in the current process could find application in several areas. For instance, it can be used as reinforced materials to obtain nanocomposites with improved mechanical and higher barrier properties against humidity and gases (O_2_ and CO_2_) [54]. This flexible and transparent nanomaterial can be used in food packaging applications to replace petroleum-based polymers. Moreover, chitin NF is using for food Pickering emulsion stabilization. This is due to its polycationic nature, capable of interacting with anionic proteins to stabilize the emulsion drops by hydrogen bond and hydrophobic interaction [55].

Additionally, chitin nanofibers can be used to prepare novel biological adhesives [56]. In the biomedical field, several biomaterials (e.g., films, membranes, aerogels) containing chitin nanofibers have been prepared. These were tested as a wound dressing and controlled release devices [57,58]. Furthermore, due to the antimicrobial properties of chitin nanofibers, they can be used in agriculture to protect plants against plant diseases and promote plant growth [59,60]. It is expected that the number of chitin NF applications will increase shortly, and some of them will reach the market, thus in this way triggering the commercial production of this material at a low cost.

## 3. Materials and Methods

### 3.1. Materials

Giant Humbold squid (*Dosidicus gigas*) was caught on the Chilean coast during the 2018–2019 season. Squid pen was provided by Landes Fishery Company (Concepción City, Chile) in a wet form. All reagents, HCl, NaOH, KBr, 2,6-bis(1,1-dimethylethyl)-4-methylphenol (BHT), methanol, chloroform, LiCl, and N,N-dimethylacetamide were purchased from Merk (Chile)

### 3.2. β-Chitin Isolation at Bench Scale

First, squid pens (gladius) were washed with tap water to separate the remaining protein debris. The samples were cut in pieces using a cutting mill SM 200 (Retsch GmbH, Germany) to obtain samples of particles size ranging from 1–3 cm. Pen sample (7 kg) was demineralized with 1 M hydrochloric acid (solids to solvent ratio of 1:10 *w*/*v*) at room temperature for 2 h in a steel reactor. The mixture was mechanically stirred at 600 rpm. The demineralized sample was filtered and washed with deionized water until the washing became neutral (pH 7) and dried at 60 °C for 24 h. The deproteinization procedure was carried out in the same reactor, where the solid was stirred with 1 M sodium hydroxide (solids to solvent ratio of 1:20 *w*/*v*) at 100 °C for 3 h. The isolated *β*-chitin was washed with distilled water and dried at 60 °C in a vacuum oven and then weighted to determine yield percent.

### 3.3. Characterization of Squid Pen and Isolated β-Chitin

#### 3.3.1. Water and Ash Content

Squid pen water content was determined gravimetrically. Each sample was placed in a porcelain crucible and heated at 105 °C in a Thermo oven (Thermo Fischer Scientific, Waltham, MA, USA) up to the constant weight. Later, the dried sample was placed in a muffle Thermo (Thermo Fischer Scientific, Waltham, MA, USA) and heated at 900 °C in order to determine the ash content [1]. Each procedure was performed in triplicate. The same methodology was followed for chitin samples.

#### 3.3.2. Total Protein Content

The total protein content (TPC) was measured by elemental analysis and calculated according to the following equation:

(1) $$\mathrm{TPC}\left(\%\right)\;=\;\left(N\left(\%\right)\;-\;6.9\right)\;\times \;6.25$$

where *N*% represents the percentage of nitrogen determined by elemental analysis for each sample; 6.9 corresponds to the theoretical percentage of nitrogen in fully acetylated chitin (this value was adjusted as a function of the degree of acetylation); and 6.25 corresponds to the theoretical percentage of nitrogen in proteins. All the determinations were done in triplicate.

#### 3.3.3. Chitin Content

The chitin content was estimated by the method reported by Black and Schwartz [2]. Namely, 0.5 g of sample was immersed in 50 mL of 0.1 M HCl, and the flask was heated at 100 °C for 1 h. Afterward, the flask was cooled to room temperature, and the content was centrifuged at 3500× *g* rpm for 10 min. The supernatant was separated from the precipitate, mixed with 45 mL of fresh DI water, and the resultant suspension was centrifuged again. This step was continuously repeated until the washing was no longer acidic. Then, 50 mL of 1.25 M NaOH solution was added to the chitin pulp product, and the mixture was heated at 100 °C for 1 h. At the end of this period, the mixture was centrifuged at 3500× *g* rpm for 10 min. The supernatant was carefully decanted from the precipitate, and 45 mL of fresh DI water was added, and the resultant suspension centrifuged. This process was repeated until the washings were no longer basic. Finally, the precipitate was washed twice with 50 mL acetone. The resulting precipitate was placed into a crucible and dried in the oven at 110 °C to constant weight. The residue should consist of chitin and silica present in the sample. The contents of the crucible were incinerated to constant weight in an electric muffle-furnace at a dull red heat (770 °C) until all carbonaceous matter was consumed. The crucible was cooled down and reweighed. The loss in weight was reported as chitin and compared to the original mass of the sample to examine the content of chitin.

#### 3.3.4. Total Lipid Content

Total lipid content was determined according to the established method [61], but with slight modification [62]. Squid pen (10 g) was blended in a top-drive blender for 2 min with a mixture of distilled water (4 mL), methanol (20 mL), and chloroform (10 mL). An additional amount of chloroform (10 mL) was added, and the mixture was mixed in a vortex for 30 s. Distilled water (10 mL) was added to the mixture and vortexed for 30 s. The mixture was filtered under pressure on a glass filter. The content was collected in a separating funnel and the two layers were isolated. The water-methanol layer was removed by suction and the chloroform layer was recovered and concentrated on a rotary evaporator. The solute was finally dried in a Thermo vacuum oven (Thermo Fischer Scientific, Waltham, MA, USA). During the process, a few crystals of 2,6-bis(1,1-dimethylethyl)-4-methylphenol (BHT) were added to the samples to prevent oxidation. All the lipid extracts were weighed, and the lipid content percentage was calculated.

#### 3.3.5. Infrared Spectroscopy Analysis

Fourier transform infrared (FTIR) spectra were recorded by a Nicolet Magna FTIR spectrophotometer (Nicolet Analytical Instruments, Madison, WI, USA). The spectrophotometer is connected to a PC with OMNIC™ software (Thermo Electron Corp., Woburn, MA, USA) to process data. The samples were prepared in KBr pellets at a concentration of 2% (*w*/*w*). The transmission spectra were recorded at 4 cm^−1^ resolution and 64 scans.

#### 3.3.6. Thermogravimetric Analysis

The thermogravimetric studies were performed by a thermogravimetric analyzer Cahn-Ventron 2000 (Cahn Scientific, Irvine, CA, USA) with a microprocessor driven temperature control unit and a thermal analysis data station. The weight of the samples ranged between 5 and 10 mg. The aluminum sample pan was placed in the balance system equipment, and the temperature was raised from 25 to 550 °C at a heating rate of 10 °C min^−1^ under a N_2_ gas flow of 50 mL min^−1^. The sample pan weight was continuously recorded as a function of temperature.

#### 3.3.7. Solid-State Cross-Polarization/Magic Angle Spinning ^13^C NMR Spectroscopy (CP/MAS ^13^C NMR)

The solid-state CP/MAS ^13^C-NMR spectra of chitin samples were registered in a Bruker AMX 300 spectrometer (Bruker, Billerica, MA, USA). In all cases, 3072 scans were accumulated. The contact time was 1 ms, the repetition time 5 s, and the acquisition time was 50 ms. The internal reference (0 ppm) was 4,4-dimethyl-4-silapentane-1-sulfonic acid (DSS). The chitin degree of acetylation (DA%) was determined from the spectrum using the ratio between the intensities (*I*) of the –CH_3_ group signal and the sum of the intensities of all carbon signals from the glucopyranosic ring, according to the following equation:

(2) $$\mathrm{DA}\left(\%\right)\;=\;\left(\frac{I_{CH3}}{I_{C1}+I_{C2}+I_{C3}+I_{C4}+I_{C5}+I_{C6}/6}\right)\times 100$$

#### 3.3.8. Scanning Electron Microscopy (SEM)

Morphological analysis was performed on an ETEC autoscan Model U-1 scanning electron microscope (University of Massachusetts, Worcester, MA, USA). The samples were fixed in a sample holder and covered with a gold layer for 3 min, using an Edwards S150 sputter coater (BOC Edwards, São Paulo, Brazil).

#### 3.3.9. Reduced Viscosity Determination

The reduced viscosity of β-chitin was determined by an Ubbelohde capillary viscometer in a water bath at 25 ± 0.1 °C. First, β-chitin was dissolved in N,N-dimethylacetamide/LiCl 5 wt%, at a polymer concentration of 0.03 g d.L^−1^ at 25 °C. The relative (*η_r_*), specific (*η_sp_*), and reduced viscosities (*η_red_*) of solutions were calculated according to the following equations:

(3) ηr=ηη0

(4) $$\eta_{sp}=\eta_{r}-1$$

(5) $$\eta_{red}=\frac{\eta_{sp}}{c}=\frac{\left(\eta_{r}-1\right)}{c}$$

where *η* is the viscosity of the solution (or dispersion); *η_0_* is the viscosity of the solvent; and *c* is the (mass) concentration in g·mL^−1^. The reduced viscosity is expressed in mL·g^−1^.

#### 3.4. β-Chitin Nanofibrils (NF) Preparation at Bench Scale

**Figure 10:**
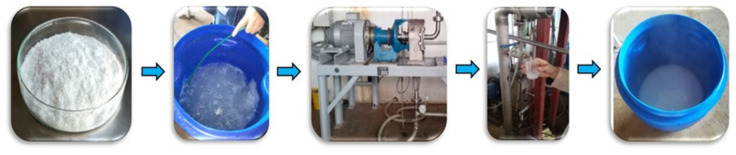
Processing of NF *β*-chitin at bench scale using a massive collider machine.

The β-chitin powder (2.1 kg) was ground in a blade mil IKA (IKA WERKLE, Staufen, Germany) and sieved up to a particle size 2 mm in diameter. The obtained solid (2 kg) was suspended in distilled water (200 L) at 1 wt%. The pH of the slurry was set up with HCl to pH 3, and the slurry was disintegrated using a proprietary massive collider operated at 25 °C (Figure 10). The *β*-chitin slurries were made to shear the fibers apart by passing the fibers through a 12-inch rotating disc. The disk gap was first set to zero, corresponding to the starting point, where the two disks graze without pulp. The mechanical defibrillation machine was operated at 1 atm pressure. The equipment loaded with a 1 wt% chitin suspension (viscosity close to cero) was fibrillated at 1200 rpm. The disintegrated β-chitin was sampled at different cycles and finally recovered. The number of collision times (=cycle pass) was set to 10, 20, 40, and 60 passes. The obtained nanofibrous material was in the form of a concentrated suspension with a gel-like appearance. Figure 10 shows the NF β-chitin processing at a bench scale.

### 3.5. Physicochemical Characterization of β-Chitin Nanofibrils

#### 3.5.1. Viscosimetry

The viscosity of the nanofibrillated gels was measured in a rotational viscometer (Fungilab, Barcelona, Spain) at 25 °C using a TL7 spin for all samples.

#### 3.5.2. Transmission Electron Microscopy (TEM)

The chitin nanofibrillated samples were diluted to obtain a 0.01 wt% solution. The solution drops were placed in a Cu grid of 100 mesh and dried at room temperature. Then, the samples were analyzed by JEOL JEM 1200 EX II TEM equipment (JEOL, Tokyo, Japan).

#### 3.5.3. X-ray Diffraction (XRD)

XRD diffractograms of chitin samples were obtained in order to evaluate their crystallite size and crystalline index. This analysis provided information about the changes in the crystalline structure of differently processed chitin nanofibrils. The XRD analysis was performed by a Bruker AXS model D4 Endeavor diffractometer (Bruker AXS GmgH, Karlsruhe, Germany) using monochromatic CuKα radiation (λ = 0.15418). The device generated a signal at 40 kV and 20 mA. The intensities were measured in the range of 5° < 2θ < 40 °C for all samples, with a step size of 0.02° and scans at one s/step. The intersheet distance was determined by Equation (6). The apparent crystallite size (Dap) was calculated using the Scherrer equation (Equation (7)), while the crystalline index (CrI) was calculated using Equation (8):

(6) n×λ=2×d×sin θ

where *n* is the order of reflection; *λ* is the radiation wavelength (in nm); and *θ* is the plane angle.

(7) $$\mathrm{Dap}=\frac{K\times \lambda }{\beta \cos\theta }$$

where *β* (in radians) was the half-width of the reflection; *K* was a constant, indicating the crystallite perfection, equal to 0.9; *λ* was the radiation wavelength (in nm); and *θ* was the plane angle.

(8) $$\mathrm{CrI}=\frac{A_{Cryst}\times 100}{A_{Total}}$$

where *A_Cryst_* was the sum of all crystalline signals and *A_Total_* was the total area of the diffractogram.

### 3.6. Preparation of Nanofibrous β-Chitin Films

In order to obtain the nanofibrillated films of different thicknesses (0.02 and 0.05 mm), different volumes of concentrated nanofibrous chitin suspension (1 wt%) were placed in Petri dishes and dried in a Thermo vacuum oven (Thermo Fischer Scientific, Waltham, MA, USA) at 60 °C for 24 h.

### 3.7. Characterization of Films

The obtained nanofibrous *β*-chitin films were subjected to XRD and SEM analysis, as described in Section 3.3.8 and Section 3.5.3, respectively.

#### Mechanical, Colorimetric, and Swelling Degree Analysis

Mechanical analysis was performed on a universal testing machine SmarTens 005 (KARG Industrietechnik, Krailling, Germany), with a load cell of 1 kN and at 23 ± 2 °C, 45 ± 5% RH. The tensile test was performed on nanofibrous β-chitin film with a width of 5 mm and length of 3 cm. The crosshead speed was 2 mm/min. All mechanical analyses were carried out in triplicate.

In order to evaluate the color changes of the β-chitin nanofiber films of different thicknesses, colorimetric analysis was performed by a Biobase BCM-200 colorimeter (Biobase Meihua Co, Jinan, China). Two measurements (center and border) were taken on each sample. Colorimetric parameters were obtained using the CIELAB color scale to measure color: L* = 0 (black) to L* = 100 (white); −a* (greenness) to +a* (redness); and −b* (blueness) to + b* (yellowness). A white (L_0_* = 94.3; a_0_* = −0.9; b_0_* = −0.7) standard color was used for equipment calibration. The color difference (Δ*E*) was calculated according to the following equation:

(9) $$\Delta E^{*}=\sqrt{{\left(L_{0}{}^{*}-L^{*}\right)}^{2}+{\left(a_{0}{}^{*}-a^{*}\right)}^{2}+{\left(b_{0}{}^{*}-b^{*}\right)}^{2}}$$

In order to check the swelling degree of chitin, the film samples were cut into 1 × 1 cm^2^ slices, and then the samples were kept in a desiccator with silica-gel for seven days. After this procedure, the samples were weighed and then subjected to immersion in glass vessels containing 10 mL of different buffer solutions (pH4, pH6, and pH8). After 24 h, samples were removed, put on tissue paper, weighed, and analyzed/recorded by a camera. The swelling degree (SD%) was calculated by Equation (10):

(10) $$\mathrm{SD}\left(\%\right)=\frac{m_{t}-m_{0}}{m_{0}}*100$$

where *m_0_* and *m_t_* are the initial weight and weight in a specific time interval.

## 4. Conclusions

This work presented the efficient extraction of β-chitin from marine-waste, squid pens of *Dosidicus gigas*. The β-form of chitin was confirmed by FTIR and NMR analysis. The degree of acetylation was 96%, as evaluated by NMR. Successful conversion of β-chitin into nanofibers at a semi-industrial scale was carried out in a collider machine under acidic conditions, which XRD, SEM, and TEM confirmed. It was shown that the number of passes of chitin through the collider machine could significantly influence the nanometer scale of the fibers. Namely, after 10 passes through the collider machine, the obtained β-chitin was in the form of visible microfibrils and nanofibrils. On the other hand, above 20 passes, the conversion of β-chitin to nanofibers was significantly improved, and the obtained nanofibers were in the range between 10 and 15 nm. The obtained nanofibers after the collider machine were in the gel-like form and were further converted into films by the solvent-casting method. Due to moderate swelling degree and good mechanical resistance, these β-chitin nanofibrous films have the potential to be further developed into food packaging, agricultural, wound dressing, or 3D-bioink material. Hence, the present work demonstrated a promising approach of utilization and industrial conversion of marine waste to β-chitin functional material with versatile potentials.

## Figures and Tables

**Table 1:** Chemical characterization of *D. gigas* squid pen and β-chitin.

Sample	Humidity (%)	Ashes (%)	Lipid (%)	Chitin (%)	Protein (%)	DA (%) ^1^	Ref
Squid pen	23.3 ± 1.2	1.8 ± 0.2	0.4 ± 0.1	30.2 ± 0.5	67.5 ± 0.4	-	Present work
*Loligo vulgaris*	-	1.7 ± 0.1	0.32 ± 0.04	40.0 ± 1.5	36.5 ± 0.6	-	[27]
*Loligo chenisis*	-	1.4 ± 0.2	1.9 ± 0.2	35.8 ±1.8	57.2 ±2.3	-	[12]
*Todarodes pacifica*	-	0.8 ± 0.1	0.20 ± 0.01	25.5 ± 0.4	74.6 ± 0.3	-	[26]
*Illex argentinus*	-	0.1 ± 0.1	2.3 ± 0.2	31.0 ± 0.6	64.0 ± 0.7	-	[11]
*β*-Chitin	10.1 ± 0.9	0.7 ± 0.2	-	-	0.7 ± 0.1	96.4	Present work

^1^ Determined by ^13^C NMR.

**Table 2:** Thermal analysis results from squid pen and *β*-chitin decomposition process.

Sample	Temperature (°C)			Weight Loss (%)
	T_Onset_	T_max_	T_End_	
Squid pen	25	46	126	23.0
	127	223	264	22.8
	264	294	550	20.2
β-Chitin	25	86	193	3.6
	193	325	550	59.4

**Table 3:** X-ray diffraction (XRD) analysis of β-chitin samples.

Sample	*d*-Spacing (Å)		D_ap_ (nm)		CI (%)
	(010)	$(1\bar{1}$0)	(010)	$(1\bar{1}$0)	
β-chitin powder	10.76	4.59	6.00	2.60	69.7
NF β-chitin film	10.05	4.48	4.70	2.60	68.0

**Table 4:** Mechanical, swelling degree, and colorimetric parameters of β-chitin nanofiber films.

Thickness (mm)	E (GPa)	TS (MPa)	e (%)	Swelling Degree (%)			CIELAB			
				pH 4	pH 6	pH 8	L*	a*	b*	ΔE*
0.02	0.9	3.94	3.46	294 ± 12	291 ± 15	257 ± 16	91.88	−0.273	1.647	3.43
0.05	1.1	17.4	1.74	287 ± 14	283 ± 12	263 ± 13	91.34	−0.226	1.712	3.87

## Data Availability

The data presented in this study are available inside the manuscript.
